# Improvement of the clinical skills of nurse anesthesia students using mini-clinical evaluation exercises in Iran: a randomized controlled study

**DOI:** 10.3352/jeehp.2023.20.12

**Published:** 2023-04-06

**Authors:** Ali Khalafi, Yasamin Sharbatdar, Nasrin Khajeali, Mohammad Hosein Haghighizadeh, Mahshid Vaziri

**Affiliations:** 1 Department of Anesthesiology, School of Allied Medical Sciences, Ahvaz Jundishapur University of Medical Sciences, Ahvaz, Iran; 2 Educational Development Center, Ahvaz Jundishapur University of Medical Sciences, Ahvaz, Iran; 3 Department of Biostatistics and Epidemiology, School of Health, Ahvaz Jundishapur University of Medical Science, Ahvaz, Iran; 4 Department of Anesthesiology, School of Medicine, Golestan Hospital, Ahvaz Jundishapur University of Medical Sciences, Ahvaz, Iran; Hallym University, Korea

**Keywords:** Anesthesia, Clinical competence, Iran, Nurse, Personal satisfaction

## Abstract

**Purpose:**

The present study aimed to investigate the effect of a mini-clinical evaluation exercise (CEX) assessment on improving the clinical skills of nurse anesthesia students at Ahvaz Jundishapur University of Medical Sciences, Ahvaz, Iran.

**Methods:**

This study started on November 1, 2022, and ended on December 1, 2022. It was conducted among 50 nurse anesthesia students divided into intervention and control groups. The intervention group’s clinical skills were evaluated 4 times using the mini-CEX method. In contrast, the same skills were evaluated in the control group based on the conventional method—that is, general supervision by the instructor during the internship and a summative evaluation based on a checklist at the end of the course. The intervention group students also filled out a questionnaire to measure their satisfaction with the mini-CEX method.

**Results:**

The mean score of the students in both the control and intervention groups increased significantly on the post-test (P<0.0001), but the improvement in the scores of the intervention group was significantly greater compared with the control group (P<0.0001). The overall mean score for satisfaction in the intervention group was 76.3 out of a maximum of 95.

**Conclusion:**

The findings of this study showed that using mini-CEX as a formative evaluation method to evaluate clinical skills had a significant effect on the improvement of nurse anesthesia students’ clinical skills, and they had a very favorable opinion about this evaluation method.

## Graphical abstract

[Fig f2-jeehp-20-12]

## Introduction

### Background/rationale

Nurse anesthetists play an essential role in providing anesthesia care and preventing anesthesia-related complications, which range from minor complications to complications that lead to morbidity and mortality [1]. Because the nature of clinical education presents challenges that may cause students to experience stress, it is difficult to teach professional career skills to students who will be medical staff in the future. In addition, learning several clinical interventions and various skills during clinical practice can create stressful situations that increase students’ anxiety and impair their learning process in the hospital [2,3]. Formative assessments are regarded as among the most important mechanisms to improve learning because their primary purpose is to facilitate learning by identifying the strengths and weaknesses of learners. However, the principled implementation of assessment methods is challenging for clinical educators. The main reason is the need for a clear definition of the constructs and objective measurement criteria to be assessed. This need has led to diversity and inconsistency in evaluation methods due to educators’ different levels of knowledge and skills [4]. Therefore, evaluating students’ competence based on well-defined standards is necessary to ensure students’ accurate performance [5]. Accurate and constructive evaluations of students with appropriate scientific methods, such as mini-clinical evaluation exercises (CEX), can reduce teaching and learning problems. According to this model, a skilled person observes the trainees’ performance according to a scaled checklist related to the target area and gives them feedback. Due to its repeatable nature, mini-CEX increases the student’s motivation to learn, and they find it to be a useful method for learning. After completing the assessment and receiving feedback, learners can identify their weaknesses and regularly review each assessed area to improve their performance [2]. In addition to evaluating clinical skills, this method also improves student-evaluator interactions. A high satisfaction level of trainees, trainers, and evaluators has also been reported when using this evaluation method [6].

### Objectives

The present study aimed to investigate the effect of mini-CEX on improving the clinical skills of nurse anesthesia students at Ahvaz Jundishapur University of Medical Sciences (AJUMS). Specifically, it assessed nurse anesthesia students’ patient management skills from when patients are prepared for general anesthesia to their emergence from anesthesia when transferred to the recovery room. It was hypothesized that mini-CEX as a formative evaluation method would improve students’ clinical skills compared to conventional methods.

## Methods

### Ethics statement

This study was approved by the Ethics Committee of AJUMS (IR.AJUMS.REC.1401.441). Informed consent was obtained from participants.

### Study design

This randomized controlled study was described according to the CONSORT (Consolidated Standards of Reporting Trials) statement (available at: https://www.consort-statement.org/).

### Setting

This study was conducted at 5 university hospitals affiliated with AJUMS and lasted for 4 weeks, from November 1, 2022, to December 1, 2022. The students’ clinical skills were evaluated 4 times during the 5th semester of their 3rd academic year. Pre- and post-tests were used to evaluate the effectiveness of mini-CEX in improving students’ skills. Before implementing the intervention, a pre-test was given to both groups of students (intervention and control). After implementing the last formative assessment using the mini-CEX in the intervention group, the students of both groups completed a post-test.

### Interventions

First, students who were homogeneous regarding their scores and clinical performance were randomly divided into intervention and control groups. A clinical competence assessment test was given as a pre-test to both groups. Students in the intervention group underwent formative assessments using the mini-CEX method (Supplement 1). Different evaluators directly observed the students’ performance from the moment the patient entered the operating room and was prepared for general anesthesia to when the patient emerged from anesthesia and was transferred to the recovery room. They were given verbal feedback, which was recorded in an evaluation form.

After 1 week, the students were re-evaluated. Each student in the intervention group was assessed once a week for 4 weeks. During this period, the control group was evaluated based on the conventional method—that is, general supervision by the instructor during the internship and summative evaluation based on a checklist at the end of the course. In the last formative assessment based on the mini-CEX, the post-test was administered again to both groups, and the intervention group filled out a mini-CEX satisfaction questionnaire.

### Participants

All 50 nurse anesthesia students were invited to participate in this study. Only the data of the students who met the inclusion criteria (5th-semester nurse anesthesia students who completed the pre- and post-tests) were included in the study. The exclusion criteria were incomplete responses to questionnaires, absence from any of the clinical practice sessions, and withdrawal from continuation of the study.

### Outcomes

The outcomes of this study included the clinical skills of students before and after the mini-CEX assessments, the clinical skills of students before and after clinical training based on the conventional method, and the students’ satisfaction with mini-CEX. The unit of analysis was the group (intervention or control).

### Data sources/measurement

The Clinical Evaluation Instrument, whose items were relevant to the study’s objectives, was selected for pre- and post-tests. This tool was developed in 2014 by Collins and Callahan [7] to evaluate the clinical skills of nurse anesthetists (Supplement 2). This tool was implemented with 137 first- and last-semester nurse anesthesia students in the United States in 1 academic semester. The instrument includes 16 items scored based on a 4-point Likert scale, with its minimum and maximum scores being 16 and 64, respectively. This tool is used to assess the following: students’ ability to assess patients in the comprehensive care of patients receiving anesthesia, ability to transfer learned content to clinical practice, management of patients before anesthesia, communication skills, professional role, and equipment preparation. The initial section of the tool collected demographic information, including age and gender. Construct validity was done for this tool [7]. In our study, the instrument was translated into Farsi by the researcher and a fluent English translator. It was then back-translated into English by an English translator who had yet to be involved in the initial translation of the instrument. To test the reliability of this tool, 15 students who met the inclusion criteria completed the form, and a Cronbach’s α coefficient of 0.854 was obtained. The evaluation form was provided to several educational professors, clinical evaluators, and 10 students who were eligible to enter the study to check face validity.

A checklist was used to implement the intervention (formative assessment of students based on the mini-CEX method). The mini-CEX formative assessment checklist was adapted from the assessment checklist of the Australian and New Zealand College of Anaesthetists [8], according to which students are assessed in 12 different areas in anesthesia based on a 9-point scoring scale (Supplement 3).

A satisfaction questionnaire was used to evaluate students’ satisfaction (supplement 4). The content validity of the questionnaire was confirmed by giving it to several experienced clinical professors and seeking their opinions. The reliability of the questionnaire was confirmed by obtaining a Cronbach’s α coefficient of 0.88 [9]. Raw response data of participants to each pre- and post-test item are available in Dataset 1. Raw response data of the participants to satisfaction survey items are available in Dataset 2.

### Bias

Because there was a possibility that different evaluators may conduct the evaluations in different sessions, short meetings were held for all evaluators participating in the study, briefing them on the mini-CEX evaluation form and how to implement it, and an already completed form was provided to the evaluators.

### Study size

Based on a previous study [10], the sample size was calculated using G*Power ver. 3.1.9.2 (Heinrich-Heine-Universität Düsseldorf), based on the independent-sample Student t-test, a 2-tailed alpha of 0.05, a power (1–β) of 0.8, and a large effect size of 0.8. The results showed that a sample size of 25 per group was required. Our study included 25 students in the control group and 25 in the intervention group.

### Randomization

All 5th-semester nurse anesthesia students who participated in the study were selected using the census method and were randomly assigned to the intervention and control groups. Group allocation was conducted by having students randomly choose numbers that were placed inside a bowl. The first number drawn out of the bowl was for the intervention group, and the second was for the control group, and this procedure continued until the last student was assigned to their group.

### Blinding (masking)

No blinding was done.

### Statistical methods

IBM SPSS ver. 25.0 (IBM Corp.) was used for data analysis. The Kolmogorov-Smirnov test confirmed the normality of the data distribution. To compare the scores in the intervention and control groups, the paired t-test, 1-way analysis of covariance, and descriptive statistics were used. The significance level was set at P<0.05.

## Results

### Participant flow

**Fig. 1.**
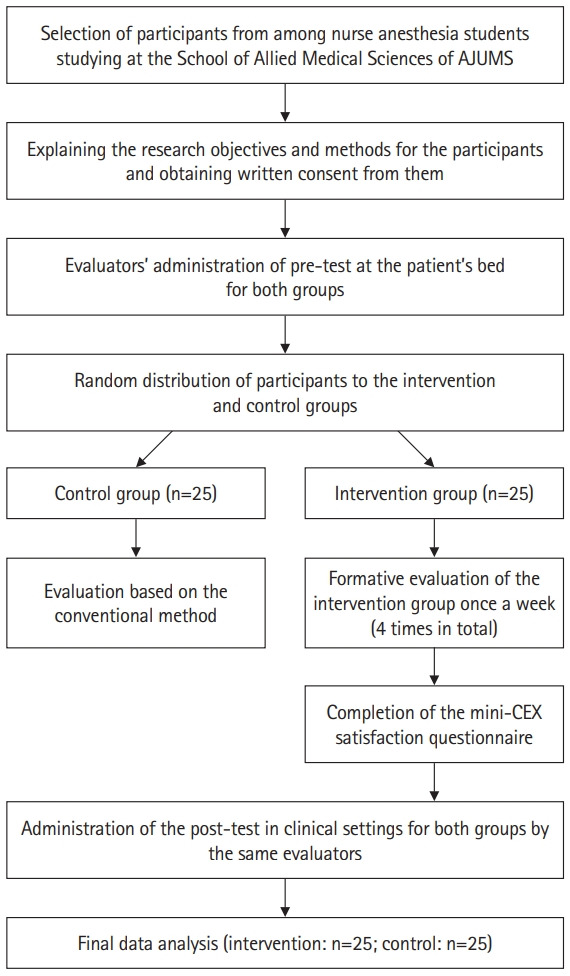
Flowchart of the study. AJUMS, Ahvaz Jundishapur University of Medical Sciences; mini-CEX, mini-clinical evaluation exercise.

All 50 nurse anesthesia students, including 13 men (26%) and 37 women (74%) with a mean age of 23±2.8 years, participated in this study. The study flow diagram is presented in Fig. 1.

### Main results

#### Clinical skills of nurse anesthesia students

The mean score of the students on the post-test increased significantly compared to the pre-test in both the control and intervention groups (P<0.0001). There was also a statistically significant difference between the mean post-test scores of the intervention and control groups (P<0.0001) (Tables 1, 2). Furthermore, as shown in Table 3, when controlling the effect of the auxiliary variable (pre-test) on the dependent variable, there was a significant difference between the 2 groups in terms of management skills (P<0.05).

#### The intervention group’s satisfaction with the mini-CEX formative assessment

Since mini-CEX had not been previously used for nurse anesthesia students in AJUMS, after the end of the intervention, the satisfaction of the intervention group students with the mini-CEX program was measured. The overall mean score of the questionnaire was 76.3 out of a maximum of 95, indicating high satisfaction with using mini-CEX in teaching and improving clinical skills. In 7 dimensions, the mean scores were very close to the maximum possible score, indicating complete satisfaction with mini-CEX. The highest level of satisfaction was related to the dimensions of fairness and improving skills (Table 4).

## Discussion

### Key results

This study investigated the effects of mini-CEX assessments on improving nurse anesthesia students’ clinical skills. The improvement of the intervention group’s clinical skills was more significant than that of the control group. Furthermore, students’ satisfaction with mini-CEX for formative assessments and education was high. Therefore, it can be concluded that the use of mini-CEX had a significant effect on improving students’ clinical skills.

### Interpretation

The study results showed that using mini-CEX for formative assessments can improve nurse anesthesia students’ clinical skills. According to the students’ scores, the mini-CEX assessments helped students master their skills and better identify their weaknesses and strengths. The students were also highly satisfied with using this method for clinical settings because they received feedback in a real environment immediately after performing these skills. In addition, the educational tips and skills resulting from this feedback were better retained by the students. This feedback is more likely to be incorporated subsequently in a similar situation than when students receive feedback only in the classroom or at the end of an internship, which again confirms the effect of the mini-CEX model on the improvement of students’ clinical skills.

In our study, students’ performance was measured objectively and under various conditions so that the evaluators could better understand the students’ performance. The summative evaluations of the students’ clinical performance could have been fairer. This result highlights the importance of using formative assessments to educate students in clinical settings better, help them acquire practical skills, develop professional responsibility, and shift from dependent and supervised practice to fully independent practice. Since the quality of education is usually measured based on the results of learners’ performance, it behooves policymakers and managers, who are involved in students’ clinical education, to adopt appropriate strategies for better education and evaluation. Therefore, using innovative and workplace-based models, such as mini-CEX, for clinical training and evaluation is necessary because the regular evaluation of students’ performance is closely related to their skill acquisition [11].

### Comparison with previous studies

The present study’s findings align with a study in India on the use of mini-CEX as a method to evaluate the clinical skills of anesthesia graduates. Most of the students and professors participating in that study had favorable opinions about various aspects of mini-CEX, such as its easy implementation and positive educational impact [12]. Another study was conducted on the perspective of trainees toward mini-CEX. According to the results of that study, 81% of learners and 75% of teachers confirmed the effectiveness of immediate feedback in the mini-CEX. Learners stated that mini-CEX was very effective in reflecting and improving their performance and learning and can be used as an educational framework for teaching and learning. Their study expressed no negative attitude toward mini-CEX [13]. The results of a similar study on the impact of the mini-CEX on the clinical competence of nursing students in Tehran were also consistent with ours. The intervention group students trained using the mini-CEX method obtained higher scores than the control group in an evaluation portfolio [2].

### Limitations

Since the evaluation was performed in clinical settings, it was only possible to partially homogenize the complexity of the clinical cases. Carrying out further studies with a larger sample size and more extended follow-up periods can shed more light on the effectiveness of this evaluation method.

### Generalizability

The results of this study have practical implications for clinical educators to better train and evaluate students in clinical settings.

### Suggestions

Anesthesia nurses are an indispensable component in anesthesia. Therefore, their efficient training and accurate assessment are of paramount importance. Therefore, future studies are recommended to compare the effectiveness of the mini-CEX against other evaluation methods.

### Conclusion

Using the mini-CEX for the formative assessment of students in clinical settings had a significant effect on improving their skills. Therefore, to teach clinical skills, evaluate students’ performance, and better understand their strengths and weaknesses in clinical settings, this model is recommended as a low-cost and effective modality along with other educational methods.

## Figures and Tables

**Figure f2-jeehp-20-12:**
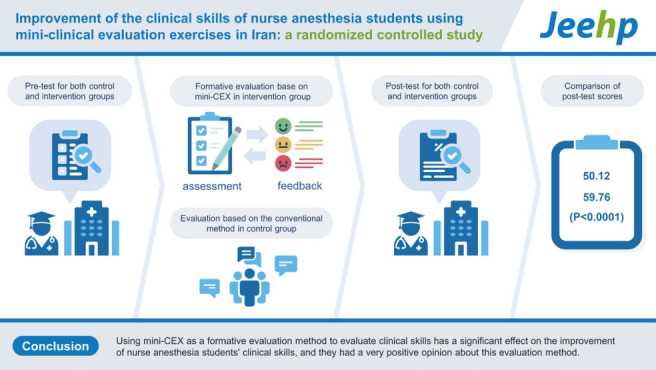


**Table 1.** Improvement of nurse anesthesia students’ clinical skills based on the mini-clinical evaluation exercise method as opposed to the conventional method

Groups	Mean±SD	P-value
Intervention		<0.0001
Pre-test	49.60±6.23	
Post-test	59.76±4.27	
Control		<0.0001
Pre-test	48.20±3.39	
Post-test	52.12±3.85	

SD, standard deviation.

**Table 2.** Comparison of post-test scores between the intervention and control groups

Groups	Mean±SD	P-value
Pre-test		0.328
Intervention	49.60±6.23	
Control	48.20±3.39	
Post-test		<0.0001
Intervention	59.76±4.27	
Control	52.12±3.58	

SD, standard deviation.

**Table 3.** Results of 1-way analysis of covariance for clinical skills

Component	Source of variation	F	P-value	Eta^2^
Management skills	Pre-test	3.030	0.005	0.69
	Group	1.652	0.005	0.75

**Table 4.** Mean scores, standard deviation, maximum score, and mean scores in the 8 dimensions of the satisfaction questionnaire

Dimension	Mean±SD	The maximum score possible in each dimension
Fairness	4.32±0.69	5
Compliance with educational goals	8.52±1.87	10
Appropriateness	13.00±1.58	15
Implementability	4.00±1.22	5
Improving skills	30.24±3.73	35
Objectivity	4.20±0.95	5
Unstressful conditions	2.88±1.12	5
Interest in using the method	11.76±2.83	15

SD, standard deviation.

## Footnotes

**Data availability**
Data files are available from Harvard Dataverse: https://doi.org/10.7910/DVN/YRL2AO Dataset 1. Raw response data of participants to each item of the pre- and post-test.

Dataset 2. Raw response data of the participants to satisfaction survey items.

## Supplementary materials

Supplementary files are available from Harvard Dataverse: https://doi.org/10.7910/DVN/YRL2AO

Supplement 1. Items that were evaluated in the formative evaluations based on mini-clinical evaluation exercise.

Supplement 2. Pre- and post-test questionnaire of the Clinical Evaluation Instrument.

Supplement 3. A checklist to implement the intervention and formative assessment of students based on the mini-clinical evaluation exercise method.

Supplement 4. Items of the satisfaction survey.

Supplement 5. Audio recording of the abstract.
