# Supplementary material for: Improvement of the clinical skills of nurse anesthesia students using mini-clinical evaluation exercises in Iran: a randomized controlled study
Source: J Educ Eval Health Prof. 2023 Apr 6;20:12. doi: 10.3352/jeehp.2023.20.12 (PMC10209614; doi:10.3352/jeehp.2023.20.12)
Supplement: Supplementary file 3 — Supplement 1. Items that were evaluated in the formative evaluations based on mini-clinical evaluation exercise. [file jeehp-20-12-suppl1.docx]

**Supplement 1.** Items that were evaluated in the formative evaluation based on mini-clinical evaluation exercise

| 1 | Basic/appropriate clinical knowledge such as airway anatomy, Mallampati score |
| --- | --- |
| 2 | Complete evaluation of the patient’s physical condition and appropriate history taking |
| 3 | Preparation of drugs for induction and reverse |
| 4 | Checking anesthesia equipment |
| 5 | Appropriate patient monitoring |
| 6 | Organization of devices for induction of anesthesia and venipuncture |
| 7 | Proper positioning of the patient for masking, intubation, extubation, and appropriate maneuvers to maintain the airway |
| 8 | Choosing the right equipment for extubation and the right catheter size for suctioning the endotracheal tube and the patient's mouth |
| 9 | Observance of sterilization protocols at work |
| 10 | Correct communication with patients and colleagues in the operating room |
| 11 | Proper technical skills for establishing an intravenous line |
| 12 | Proper technical skills for masking and patient ventilation |
| 13 | Proper technical skills for creating a safe airway and fixing an endotracheal tube |
| 14 | Proper technical skills in weaning the patient from the ventilator |
| 15 | Proper technical skills for correct patient suction and endotracheal tube |
| 16 | Proper technical skills for the correct transfer of patient to the recovery bed |
