# Supplementary material for: Improvement of the clinical skills of nurse anesthesia students using mini-clinical evaluation exercises in Iran: a randomized controlled study
Source: J Educ Eval Health Prof. 2023 Apr 6;20:12. doi: 10.3352/jeehp.2023.20.12 (PMC10209614; doi:10.3352/jeehp.2023.20.12)
Supplement: Supplementary file 4 — Supplement 2. Pre- and post-test questionnaire of the Clinical Evaluation Instrument. [file jeehp-20-12-suppl2.docx]

**Supplement 2.** Pre- and post-test questionnaire of Clinical Evaluation Instrument

| **I. Patient assessment and anesthetic plan**  1. Consistently performs a thorough preoperative and postoperative evaluation on each patient as appropriate  Above expectations  Meets expectations  Below expectations  Failing  2. Synthesizes a comprehensive care plan for patients in all American Society of Anesthesiologists physical status categories  Above expectations  Meets expectations  Below expectations  Failing |
| --- |
| **II. Didactic transference to clinical practice**  1. Consistently utilizes critical thinking skills in applying didactic knowledge to clinical cases  Above expectations  Meets expectations  Below expectations  Failing |
| **III. Perianesthetic management**  1. Uses sound clinical judgment when managing routine, advanced, and emergency cases  Above expectations  Meets expectations  Below expectations  Failing  2. Readily achieves mastery of new skills and procedures  Above expectations  Meets expectations  Below expectations  Failing  3. Synthesizes perioperative data to make safe adjustments in care  Above expectations  Meets expectations  Below expectations  Failing  4. Serves as a resource person for airway and ventilatory management of patients  Above expectations  Meets expectations  Below expectations  Failing  5. Recognizes and appropriately responds to complications that occur in the perioperative period  Above expectations  Meets expectations  Below expectations  Failing |
| **IV. Communication skills/professional role**  1. Demonstrates efficiency  Above expectations  Meets expectations  Below expectations  Failing  2. Validates and critiques own performance  Above expectations  Meets expectations  Below expectations  Failing  3. Independently communicates with all anesthesia, operating room (OR), and surgical personnel  Above expectations  Meets expectations  Below expectations  Failing  4. Treats patients respectfully  Above expectations  Meets expectations  Below expectations  Failing  5. Stress management is appropriate  Above expectations  Meets expectations  Below expectations  Failing |
| **V. Care and preparation of equipment**  1. Works within the budgetary and accreditation goals of the OR/anesthesia department  Above expectations  Meets expectations  Below expectations  Failing  2. Identifies and takes appropriate action when confronted with equipment-related malfunctions  Above expectations  Meets expectations  Below expectations  Failing  3. Follows standard precautions for safety and infection control  Above expectations  Meets expectations  Below expectations  Failing |
