# Supplementary material for: Improvement of the clinical skills of nurse anesthesia students using mini-clinical evaluation exercises in Iran: a randomized controlled study
Source: J Educ Eval Health Prof. 2023 Apr 6;20:12. doi: 10.3352/jeehp.2023.20.12 (PMC10209614; doi:10.3352/jeehp.2023.20.12)
Supplement: Supplementary file 5 — Supplement 3. A checklist to implement the intervention and formative assessment of students based on the mini-clinical evaluation exercise method. [file jeehp-20-12-suppl3.docx]

**Supplement 3.** A checklist to implement the intervention, formative assessment of students based on the mini-clinical evaluation exercise method

| **Date of assessment: Trainee name: Assessor name:** | |
| --- | --- |
| **Areas for assessment** | **Site to record assessor’s feedback** |
| **Knowledge** | Does trainee demonstrate relevant basic science/clinical knowledge and understanding pertaining to the case? |
| **Patient assessment** | Does trainee perform a complete and appropriate assessment of the patient and present well documented findings? |
| **Planning** | Does trainee formulate an appropriate clinical plan demonstrating an understanding of relevant issues related to the patient, procedure, pathology, positioning, place, and so forth? |
| **Preparation** | Does trainee prepare appropriately for any intervention, check equipment, organize theatre and monitoring, prepare drugs, and ensure appropriate personnel are present? |
| **efficiency** | Does trainee create a well-organized workspace, use time effectively and efficiently? |
| **Consciousness** | Does trainee demonstrate situational awareness through constant monitoring of the patient (clinically and electronically), the procedure and other personnel? |
| **Problem solving and decision making** | Does trainee demonstrate sound judgment and clinical decision making? |
| **Insight** | Does trainee recognize limits of their expertise and experience? Does trainee take on responsibility appropriately? |
| **Technical skill** | Does trainee demonstrate proficiency? (Including proper positioning of the patient for masking, intubation, extubation, appropriate maneuvers to maintain the airway, inserting IV-line, etc.) |
| **Patient interaction** | Does trainee develop rapport and trust; accurately elicit, synthesize, and convey relevant information; develop a common understanding of issues, problems, and plans? |
| **Team interaction** | Does trainee participate effectively and appropriately in an inter-professional healthcare team? |
| **Risk management** | Does trainee practice to reduce medical error; comply with hospital and college protocols and guidelines? |
