# Supplementary material for: Improvement of the clinical skills of nurse anesthesia students using mini-clinical evaluation exercises in Iran: a randomized controlled study
Source: J Educ Eval Health Prof. 2023 Apr 6;20:12. doi: 10.3352/jeehp.2023.20.12 (PMC10209614; doi:10.3352/jeehp.2023.20.12)
Supplement: Supplementary file 6 — Supplement 4. Items of the satisfaction survey. [file jeehp-20-12-suppl4.docx]

**Supplement 4.** Items of satisfaction survey

|  | | | **Satisfaction** | | | | |
| --- | --- | --- | --- | --- | --- | --- | --- |
|  | **Areas** | **Items of satisfaction questionnaire** | **Strongly agree** | **Agree** | **Neither agree nor disagree** | **Disagree** | **Strongly disagree** |
| **1** | **Fairness** | The grade awarded to the student in this method is fair. |  |  |  |  |  |
| **2** | **Compliance with educational goals** | The measured items in this method are in accordance with the educational objectives. |  |  |  |  |  |
|  |  | Carrying out assessment with this method does not cause disruption in the implementation of other programs and educational goals. |  |  |  |  |  |
| **3** | **Appropriateness** | This method is a suitable tool for assessing practical skills. |  |  |  |  |  |
|  |  | This method is a suitable tool for assessing interview skills and history taking. |  |  |  |  |  |
|  |  | This method is a suitable tool for assessing student's behavior and attitude. |  |  |  |  |  |
| **4** | **Implementability** | It is possible to perform assessment in this way at the patient's bedside in teaching hospitals. |  |  |  |  |  |
| **5** | **Improving skills** | Conducting evaluation with this method improves practical skills by providing feedback to students. |  |  |  |  |  |
|  |  | In this method, the student's application of what she/he has learned is evaluated, not just what she/he has memorized. |  |  |  |  |  |
|  |  | Conducting evaluation with this method will improve behavioral skills and attitude in students. |  |  |  |  |  |
|  |  | Evaluation with this method improves the student's communication skills. |  |  |  |  |  |
|  |  | Doing evaluation with this method can lead to deeper learning of clinical skills of students. |  |  |  |  |  |
|  |  | Doing evaluation with this method makes it easier for students to learn clinical skills. |  |  |  |  |  |
|  |  | Using this evaluation method creates interest in individual studies and learning in students. |  |  |  |  |  |
| **6** | **Objectivity** | This method provides an objective assessment (certainty of the steps and criteria to be assessed for each of the mentioned techniques/accurate assessment and away from general comments). |  |  |  |  |  |
| **7** | **Unstressful conditions** | This clinical evaluation method does not cause stress and anxiety in students. |  |  |  |  |  |
| **8** | **Interest in using the method** | I am interested in using this clinical evaluation method for other techniques. |  |  |  |  |  |
|  |  | I am interested in using this evaluation method for other clinical departments. |  |  |  |  |  |
|  |  | I recommend the use of this clinical evaluation method to other groups. |  |  |  |  |  |

Minimum score=19, maximum score=95. Scores between 19–38 represent completely dissatisfied, 39–57: relatively dissatisfied, 58–76: relatively satisfied, and 77–95: completely satisfied.
